# Impact of the COVID-19 Pandemic on the Epidemiology of Bloodstream Infections in Hospitalized Patients—Experience from a 4th Military Clinical Hospital in Poland

**DOI:** 10.3390/jcm12185942

**Published:** 2023-09-13

**Authors:** Natalia Słabisz, Ruth Dudek-Wicher, Patrycja Leśnik, Jacek Majda, Krzysztof Kujawa, Urszula Nawrot

**Affiliations:** 1 Department of Laboratory Diagnostic, 4th Military Clinical Hospital in Wroclaw, 53-114 Wroclaw, Poland; nataliaslabisz@gmail.com (N.S.); jmajda@4wsk.pl (J.M.); 2 Department of Pharmaceutical Microbiology and Parasitology, Faculty of Pharmacy, Wroclaw Medical University, 50-367 Wroclaw, Poland; urszula.nawrot@umw.edu.pl; 3 Clinical Department of Anesthesiology and Intensive Care, 4th Military Clinical Hospital in Wroclaw, 53-114 Wroclaw, Poland; patrycja.lesnik@gmail.com; 4 Statistical Analysis Centre, Wroclaw Medical University, 50-368 Wroclaw, Poland; krzysztof.kujawa@umw.edu.pl

**Keywords:** SARS-CoV-2, COVID-19, bloodstream infection, nosocomial infection, MDRO

## Abstract

Bloodstream infections (BSIs) are associated with high mortality and inappropriate or delayed antimicrobial therapy. The purpose of this study was to investigate the impact of the COVID-19 pandemic on the epidemiology of BSIs in hospitalized patients. The research aimed to compare the incidence of BSIs and blood culture results in patients hospitalized before and during the COVID-19 pandemic. Methods: Retrospective and prospective data were collected from blood cultures obtained from 4289 patients hospitalized between June 2018 and July 2022. Two groups of patients were distinguished: those with BSIs admitted during the pre-COVID-19 period and those admitted during the COVID-19 surge. Demographic and clinical data, blood cytology, and biochemistry results were analyzed, and the usefulness of PCT was assessed in patients with COVID-19. Results: The study showed a significant increase in the incidence of BSIs during the pandemic compared to the pre-COVID-19 period. Positive blood cultures were obtained in 20% of patients hospitalized during the pandemic (vs. 16% in the pre-COVID-19 period). The incidence of BSIs increased from 1.13 to 2.05 cases per 1000 patient days during COVID-19, and blood culture contamination was more frequently observed. The mortality rate was higher for patients hospitalized during the COVID-19 pandemic. An increased frequency of MDRO isolation was observed in the COVID-19 period. Conclusions: The incidence of BSIs increased and the mortality rate was higher in the COVID-19 period compared to the pre-COVID-19 period. The study showed limited usefulness of procalcitonin in patients with COVID-19, likely due to the administered immunosuppressive therapy.

## 1. Introduction

Bloodstream infections (BSIs) are one of the leading causes of morbidity and mortality, especially in critically ill patients. Regardless of whether the infection is community- or hospital-acquired, it complicates hospital stays, is associated with prolonged hospitalization ranging from 2 to 32 days, and is associated with increased costs attributed to BSI [1]. Bacteremia affects nearly 20% of ICU patients and has been associated with negative outcomes as a major contributor to in-hospital mortality. BSIs are defined by the presence of pathogenic microorganisms in the bloodstream, confirmed by one or more positive blood cultures. Primary BSIs occur without any defined nidus of infection, while secondary BSIs are the result of an infection caused by the same pathogen in another anatomical region, e.g., a urinary tract infection with subsequent bacteremia [2]. The diagnosis of BSI can be complicated due to blood culture contamination. The most common contaminants in blood cultures are coagulase-negative staphylococci (CoNS), which, on the other hand, are the most important cause of BSIs in patients with implanted devices and indwelling catheters (CLABSI—central line-associated BSI). Conversely, the next most frequent bacteria, *Corynebacterium* spp. and *Propionibacterium* spp., almost always represent contamination [3,4]. Blood cultures are commonly collected when patients have signs of bacteremia or sepsis and should be taken as soon as possible before the administration of antibiotics [5]. Detailed rules for the management of patients with suspected sepsis or septic shock are described in the Surviving Sepsis Campaign (SSC) guidelines [6].

The pandemic of SARS-CoV-2 infection at the beginning of 2020 has severely hit many countries in the world, causing the deaths of more than six million people worldwide by the end of 2022. In the 2020–2022 period, COVID-19 has become one of the main challenges for public healthcare, mainly due to the large number of patients requiring intensive care. Pneumonia outbreaks caused by SARS-CoV-2 can be complicated by secondary bacterial or fungal infections, which are more frequent, especially in critically ill patients treated in the ICU (31.5%) than in standard medical COVID-19 wards (9%) [7,8,9,10]. The overlap of clinical manifestations of viral and bacterial infections makes diagnosis very difficult, and consequently, the percentage of bacterial superinfections in COVID can be higher than presumed.

BSIs are another common complication in patients with severe COVID-19, with limited data available, and most studies have been conducted in critically ill patients in the ICU. It has been shown that a total of 7% of hospitalized COVID-19 patients may develop BSIs, with a mortality rate of about 40% [9]. COVID-19 patients are three times more likely to develop BSI compared to patients hospitalized for other reasons than SARS-CoV-2 infection [7]. As shown by several studies, the highest increase in incidence during the surge of COVID-19 was observed for *Acinetobacter baumannii*, carbapenem-resistant *Klebsiella pneumoniae*, *Pseudomonas aeruginosa*, methicillin-resistant *Staphylococcus aureus* (MRSA), and *Enterococcus* [11,12,13,14]. A high proportion of multi-drug-resistant organisms causing BSIs in patients with COVID-19 suggests that the overuse of antibiotics plays a major role in the selection of bacterial strains resistant to pharmacological treatment. In addition, reported statistical data were certainly influenced by healthcare service crises related to the implications of the COVID-19 pandemic, e.g., absences of medical staff, difficulties in implementation of infection prevention and control programs in hospitals, screening for the carriage of resistant pathogens, and isolation and cohorting of patients [8,9,15,16].

The aim of this study was to try to answer the question of how much the COVID-19 pandemic has affected the incidence of BSIs in patients hospitalized at the 4th Military Clinical Hospital in Wroclaw, Poland. To reach this goal, we compared the incidence of BSIs and blood culture results in patients hospitalized two years before and two years during the COVID-19 pandemic. In addition, the usefulness of CRP and PCT in diagnosing secondary BSI infections in the course of COVID-19 was assessed.

## 2. Materials and Methods

### 2.1. Study Design

The data analysis was started at the end of 2022 and was utterly retrospective. The results of blood cultures obtained from 4289 patients in the period from June 2018 to July 2022 were collected and analyzed. The subject of the analysis were the results of blood cultures and biochemical tests performed as part of routine diagnostics at the Department of Laboratory Diagnostic, 4th Military Clinical Hospital in Wroclaw, a 500-bed medical center in Wroclaw, Poland. The study protocol was approved by the Bioethics Committee of the Military Medical Chamber, Poland (approval no. 194/22).

### 2.2. Data Collections

Blood samples collected from adult hospitalized patients in the period between June 2018 and July 2022 were included in the study. Two groups of patients were distinguished. One group included patients with BSIs admitted during so-called “preCOVID-19” (June 2018 to May 2020) and the second group during the SARS-CoV-2 surge called “COVID-19” (June 2020 to July 2022). The patient’s demographic and clinical data were recorded in the electronic medical database. There are two IT systems in the hospital that communicate with each other. The master program is the AMMS system (Asseco Medical Management Solutions), designed to support medical facilities and generate electronic medical records. The cooperating system is laboratory software Marcel CENTRUM, which sends the results of all laboratory tests to the AMMS system. Demographic data such as age, sex, comorbidities (diabetes, chronic kidney disease, chronic cardiovascular disease, cancer), overweight (BMI ≥ 25), and information about the patient’s death were obtained from the AMMS database, considering the medical history. Selected data of blood cytology and biochemistry (a white blood cell count (WBC; cells × 10^3^/μL), blood neutrophil count (NEUTR; cells × 10^3^/μL), serum lactate level (LAC; mmol/L), serum C-reactive protein level (CRP; mg/L), and serum procalcitonin level (PCT; ng/mL)) from the same day as the collection of the first blood sample that resulted in a positive culture were extracted from the electronic laboratory database.

### 2.3. Definitions

BSI was defined when at least one positive blood culture for bacteria or fungi was obtained. For coagulase-negative staphylococci (CoNS) and other common skin residents (*Corynebacterium* spp., *Cutibacterium* spp.), at least two consecutive blood cultures set positive for the same pathogen were required to define BSI [17]. *Staphylococcus aureus*, *Streptococcus pneumoniae*, *Enterobacterales*, *Pseudomonas aeruginosa*, and *Candida albicans* were regarded as predictive for true BSI even if grown from a single culture bottle. Multiple positive blood cultures for the same organism in the same patient were considered one BSI episode. Contamination was defined as the detection of microbes considered non-pathogenic and likely to be introduced into the culture during specimen collection in a single blood culture bottle [3,4].

Patients were assigned to the COVID-19 group when at least one real-time polymerase chain reaction (PCR) assay positive for SARS-CoV-2 in a respiratory specimen (nasopharyngeal swab) was obtained. Patients treated due to COVID-19 within 6 months prior to hospital admission were also included in this group. In these cases, data on past infections was taken from the medical history. The patient’s death was defined as a death during hospitalization.

BSI suspicion and blood cultures. According to the guidelines of the Surviving Sepsis Campaign [6], in cases of suspicion of sepsis or septic shock, 2–3 blood cultures, collected in bottles with medium intended for the cultivation of aerobic and anaerobic organisms, should be secured each time before the administration of the antibiotic. Indications for blood culture collection include suspected bacterial endocarditis, the presence of a catheter, and a fever >39.4 °C. In the case of fever >38.3 °C, patient age >65 years, chills, vomiting, systolic blood pressure drops <90 mmHg, significant leukocytosis, or creatinine increase >2 mg/dL, blood collection for culture should be performed when a minimum of two of these factors exist. Then, within one hour of diagnosis, start empiric antibiotic therapy. If a blood infection of MRSA or MDRO etiology is suspected, e.g., related to previous carriage, broad-spectrum antibiotic therapy should be used, considering these microorganisms.

### 2.4. Microbiology Procedure

In accordance with the hospital’s procedure for collecting material for microbiological tests, two to three sets of blood specimens should be collected from independent venipuncture sites, and, for adult patients, each set (aerobic and anaerobic bottles) should consist of 20 mL of blood. Blood culture bottles BacT/ALERT FN PLUS and BacT/ALERT FA PLUS (bioMérieux, Craponne, France) were incubated in a BacT/ALERT 3D instrument (bioMérieux, France) at 37 °C for 5 days. After the growth was detected, the positive blood cultures were Gram-stained and streaked onto Columbia Agar (bioMérieux, France), Chocolate Agar (bioMérieux, France), MacConkey (bioMérieux, France) and Schaedler Agar (bioMérieux, France) for incubation in 5% carbon dioxide at 37 °C overnight. The VITEK-2 automated system (bioMérieux, France) was used for isolate identification and antimicrobial susceptibility testing. VITEK2 AST-N331 and AST-N332 (AST—antimicrobial susceptibility testing) panels were used for antibiotic susceptibility testing of Gram-negative rods, AST-P643, AST-P643, AST-ST01 for Gram-positive cocci, and AST-YS08 for fungi. Phenotypic detection of carbapenemases was performed by an immunochromatography test (RESIST-5 O.O.K.N.V, CorisBioConcept, Gembloux, Belgium). The results of the susceptibility tests were interpreted according to the current criteria of the European Committee on Antimicrobial Susceptibility Testing (EUCAST) [18]. The analysis of the drug susceptibility of strains isolated from BSI was limited to a comparison of the prevalence of the most relevant resistance mechanisms, i.e., MRSA, MRCNS, VRE, KPC, and NDM.

### 2.5. Statistics

The statistical significance of the differences in demographic data, selected blood biochemistry results, mortality, and the distribution of causative BSI pathogens between the patient groups was calculated with the use of the Mann–Whitney test (when two numerical variables were compared), the Kruskal–Wallis test (three numerical variables were compared), and Pearson’s Chi-squared test (for categorical variables). When the term “significant/significantly” is used, it refers to statistical significance at the threshold value of 0.05). The statistical analysis was performed using Statistica 13.3 (TIBCO, Software Inc., Palo Alto, CA, USA, 2017).

## 3. Results

In the analyzed period at the 4th Military Clinical Hospital in Wroclaw, more than 83,500 patients were hospitalized. Within two years prior to the outbreak of the SARS-CoV-2 virus pandemic (June 2018 to May 2020—preCOVID-19), the number of hospitalizations was 46,849, while during the pandemic (June 2020 to July 2022—COVID-19), it decreased to 36,733. Despite the decline in admissions during COVID-19, the number of individual hospitalization days in both analyzed periods was comparable—262.174 and 233.447, respectively (Table 1).

**Figure 1:**
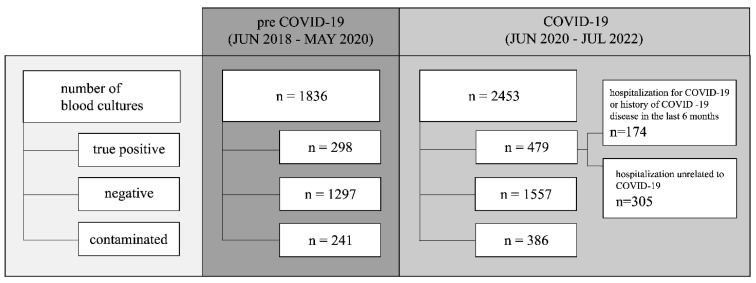
Blood cultures performed during pre-COVID-19 and COVID-19 periods.

Noteworthy, during the COVID-19 pandemic, there was a nearly 34% increase in the number of patients with indications for blood infection diagnostics. Positive blood cultures were obtained in 20% of patients hospitalized during the pandemic period compared to 16% treated in preCOVID-19, and the difference is statistically significant (χ^2^ test: df = 1, *p* = 0.005). Comparing the studied periods, the incidence of BSIs increased from 1.13 to 2.05 cases per 1000 patient-days during COVID-19. Also, in this period, blood sample contamination was observed more frequently (16% vs. 13%, χ^2^ test: df = 1, *p* = 0.016) (Figure 1).

The median age in both groups was 73 years, and the percentage of male sex was almost 57% (pre-COVID-19) and 60% (COVID-19). Both the differences in age and gender between the analyzed groups were statistically insignificant (Table 2). In both groups of patients with BSI, statistically significant differences were observed in the frequency of certain co-morbidities. During the pandemic, there were fewer patients with chronic cardiovascular disease (32% vs. 52%) but more with diabetes (31% vs. 24%) and renal failure (21% vs. 10%) (Table 2). Mortality during the COVID-19 pandemic was higher compared to previous years, especially in patients hospitalized due to SARS-CoV-2 infection (47% vs. 42%) (Table 2).

Co-morbidities such as cardiovascular disease, cancer, obesity, and chronic kidney disease were associated with a high risk of death, but in the pre-COVID-19 period, greater risk was connected with the co-existence of cardiovascular disease, while during the pandemic, it was connected with the co-existence of kidney failure. In patients hospitalized in the COVID-19 period, multiple infections were statistically more frequent, and the isolation of two species of microorganisms was associated with an increased risk of death. All the listed differences were statistically significant (Table S1).

**Figure 2:**
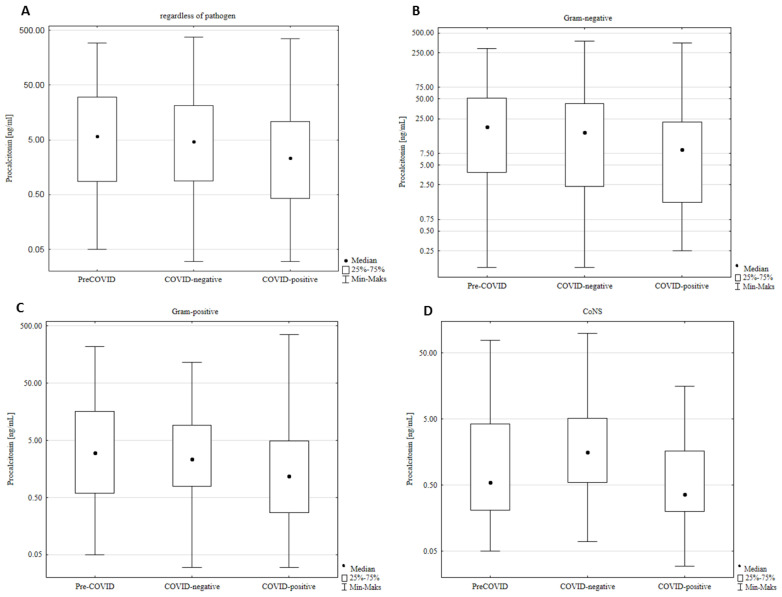
Procalcitonin (PCT) level in different patient subpopulations. A/COVID–positive patients with BSI showed a significantly lower level of PCT than patients with BSI and without SARS-CoV-2 infection, including those hospitalized in pre-COVID-19 (*p* = 0.0005) and COVID-19 pandemic periods (*p* = 0.01) (Kruskal–Wallis test) (**A**). Differences in PCT levels were also significant when patient groups with/without SARS-CoV2 infection and concomitantly infected with Gram-negative bacteria (**B**) (*p* = 0.03), Gram-positive bacteria (**C**) (*p* = 0.028; *p* = 0.002), or CoNS (*p* = 0.002) (**D**) were analyzed separately.

Among the inflammatory parameters analyzed in both groups, statistically significant differences were observed in the level of PCT. Patients with BSI that were hospitalized due to SARS-CoV-2 infection showed lower levels of PCT compared to patients without COVID-19. The level of other inflammatory parameters (WBC, NEUTR, CRP, and LAC) did not differ statistically significantly between both groups (Table 2). Additional analysis of the relationship between the production of inflammatory markers and the etiological agent of BSI (Mann–Whitney test) showed some statistically significant differences in PCT and LAC levels (Table S2). Higher values of these parameters were observed in cases of infections caused by Gram-negative rods, including both *Enterobacterales* and non-fermenting rods such as *Pseudomonas* spp. and *Acinetobacter* spp. In the case of infections caused by CoNS, the values of all inflammatory parameters were significantly lower when compared to infections caused by other etiological factors (*p* < 0.05) (Table S2). Patients with COVID-19 produced lower levels of PCT than patients without SARS-CoV-2 infection, both in relation to infections caused by Gram-negative and Gram-positive bacteria (Figure 2).

**Figure 3:**
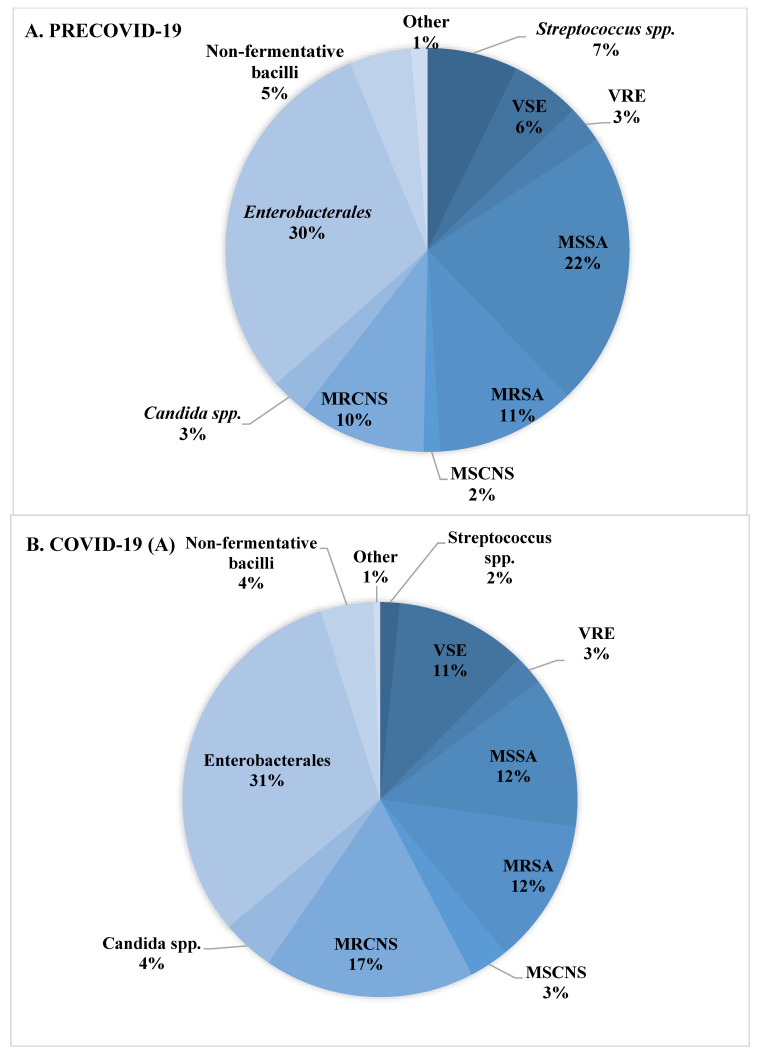

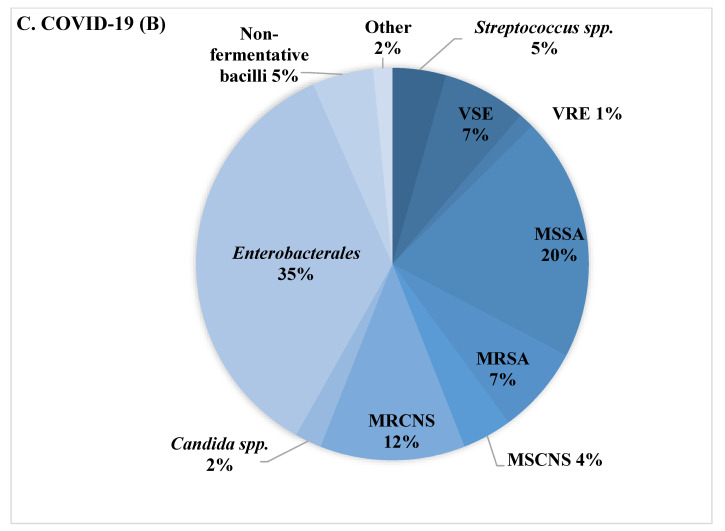
Distribution of species detected in blood cultures in patients with BSI. (**A**)—species isolated during the pre-COVID-19 period. (**B**)—species isolated during the COVID-19 period from patients with SARS-CoV-2 infection. (**C**)—species isolated during the COVID-19 period from patients without SARS-CoV-2 infection.

The distribution of species isolated from blood in the patient groups studied is shown in Table 3 and Figure 3. Significant differences were observed in the prevalence of CoNS, including *Staphylococcus epidermidis*. CoNS-caused BSIs were more frequent in patients hospitalized during the SARS-CoV-2 pandemic when compared to the pre-COVID period (17% vs. 11%) and were the highest in the subpopulation of patients hospitalized due to COVID-19 (19%). There was also a higher percentage of *Enterococcus* spp.-caused BSIs in COVID-19 patients, but these differences were not statistically significant (Table 3, Figure 3). The percentage of MRSA isolation for the two analyzed groups (preCOVID-19 and COVID-19) was the same—34%; however, significant differences in the proportion of MRSA to methicillin-sensitive *S. aureus* (MSSA) were noted. Among *S. aureus* isolated from patients with COVID-19, nearly half (49%) were MRSA, while among those isolated from patients treated for reasons other than COVID-19, the percentage of MRSA isolation was 26% (Table 4, Figure 3). A similar situation occurred in the analysis of the frequency of BSIs caused by *K. pneumoniae* NDM (New Delhi metallo-beta-lactamases). Statistically significant differences were shown both between the preCOVID-19 and COVID-19 groups (1% vs. 7%) as well as within the pandemic period, in which the percentage of patients with COVID-19 and *K. pneumoniae* NDM-caused BSIs was 16% of all Gram-negative rods, while in the case of other patients hospitalized, *K. pneumoniae* NDM-caused BSIs were responsible for 2% of BSIs (Table 4). There were no statistically significant differences in the isolation of Gram-negative rods producing extended spectrum β-lactamases (ESBL) and non-fermenting rods such as *A. baumannii*, *P. aeruginosa*, or *Stenotrophomonas maltophilia* (Table 3, Figure 3).

## 4. Discussion

In the four-year observational study, a comparative analysis of BSIs that occurred during the pre-COVID-19 and COVID-19 periods is presented.

The incidence of BSIs during the pandemic period was higher (2.05/1000 patient days) compared to pre-COVID-19 phase, which was probably associated with the hospitalization of a large number of patients suffering from COVID-19. These data are in line with other literature reports that indicate numerous risk factors predisposing patients infected with SARS-CoV-2 to secondary infections. The most important are, among others, the deregulation of immune response to viral infection through the cytokine storm (hypercytokinemia) and the reduced production of IFN-γ as a consequence of a decrease in CD4+ lymphocyte to Th1 subtype differentiation. Further, it has been shown that COVID-19 patients more often required an extension of hospitalization, including a stay in the intensive care unit (ICU), and were therefore more likely to acquire a nosocomial infection. Immunosuppressive therapy such as corticosteroids or interleukin-6 inhibitors used in the course of the disease is also crucial [16,19].

However, the calculated COVID-19 pandemic period number of BSI incidence (2.05/1000 patient-days) does not reflect the prevalence of infections only among patients hospitalized due to SARS-CoV-2 infection. This number also includes patients treated for other reasons, which is one of the limitations of this study. It was not possible to determine the number of person-days generated only by COVID-19 patients. The literature data about BSI frequency from the pandemic period often come from intensive care units only, where due to the severe condition of patients, a significantly higher index (23.56–47) was obtained per 1000 patient-days compared to our observations. The BSI incidence for the normal ward and ICU overall was close to the results obtained in our study (3.05 vs. 2.05 per 100 patient-days) [8,16,17,20]. However, the epidemiology of BSIs was similar in both studied groups, but an increased frequency of MDR (multidrug-resistant) strain isolation was observed in the case of COVID-19 patients.

Among the factors contributing to the rise in the number of healthcare-associated infections (HAI) are the transmission of hospital pathogens by medical staff and the abuse of antibiotics. Subsequent waves of COVID-19 caused an increased inflow of patients to health care institutions. Medical staff was overwhelmed with work, and staff shortages among doctors and nurses led to an increase in the number of patients per medical professional. Frequent rotation of medical staff between departments and even individual units was observed, which could have a relationship with pathogen circulation. Amid limited supply and difficult access to personal protective equipment (PPE), it was spared by medical staff. Paradoxically, PPE such as overalls, glasses, masks, or double gloves, while protecting against SARS-CoV-2 infection, contributed to the spread of MDR strains. The use of urinary catheters, central lines, and respirators increased, but concomitant HAI prevention procedures such as central injection care were neglected. This has led to a particular increase in the number of central line-associated blood stream infections (CLABSI), which has been observed in both the United States and Europe [20,21]. In this study, a statistically significant (*p* = 0.023) increase in the frequency of CoNS isolation from COVID-19 patients’ blood samples was demonstrated. The limitation of the conducted research may be the fact that, from the retrospectively analyzed medical documentation, it was not possible to obtain information on whether, in each case, the isolation of CoNS from blood was univocal with the qualification of infection as CLABSI. Due to the frequent lack of a comprehensive diagnosis of CLABSI consisting of simultaneous blood samples collected from the central injection, peripheral blood, and the tip of the removed central injection, it was not possible to classify the case as laboratory-confirmed CLABSI.

The COVID-19 pandemic has resulted in a significant increase in antibiotic consumption and, thus, in the frequency of hospital infections caused by MDR strains. The initial concerns about bacterial coinfections, a lack of evidence-based treatment options, and severe clinical conditions resulted in the inclusion of antibiotic therapy in more than 60% of patients with COVID-19 [21,22]. A particular rise in antibiotic consumption was noted for broad-spectrum antibiotics such as cephalosporins, carbapenems, and aminoglycosides [23].

In our study, a statistically significant increase in the isolation of *K. pneumoniae* NDM strains was shown in patients with BSIs hospitalized due to SARS-CoV-2 infection. Among this group, the proportion of NDM strains accounted for 16% of all isolated Gram-negative rods. In the case of patients hospitalized for reasons other than COVID-19, this percentage was 2%.

The reviews concerning the impact of the COVID-19 pandemic on the epidemiology of hospital infections and the drug resistance of microorganisms differ significantly and indicate that the incidence of secondary infections caused by carbapenem-resistant *K. pneumoniae* strains in COVID-19 patients is between 0.35 and 53%. The most commonly observed types of carbapenemases were KPC, OXA-48, and NDM, and infections were mainly related to pneumonia and BSIs. This is most likely due to mechanical ventilation and the presence of central lines, together with the neglect of nosocomial infection prevention procedures [22,24,25]. Also, broad meta-analyses confirmed MRSA as an important etiological factor of secondary infections, including ventilator-associated pneumonia (VAP) and severe blood infections, in people hospitalized for respiratory failure in the course of SARS-CoV-2 infection. Significant effects of MRSA coinfection on mortality due to COVID-19 have also been shown, especially among patients requiring hospitalization in intensive care units [22,25,26,27]. In the study conducted in 4 hospitals by Pasquini et al., it was described as having a more than seven times higher rate of MRSA isolation from BSIs in COVID-19 patients compared to those treated for other than COVID-19 conditions [16]. This observation corresponds to our results, where a statistically significant increase in MRSA isolation was noticed for patients with BSIs and COVID-19 (*p* = 0.009). MRSA was isolated from all BSI caused by *S. aureus*, almost twice as often in COVID-19 patients than non-COVID-19 patients.

Moreover, in this study, we present a high proportion of BSIs caused by *Enterococcus* spp. in patients with COVID-19. This is consistent with a retrospective cohort study of 89 patients hospitalized in the Intensive Care Unit for SARS-CoV-2 infection conducted by Bonazzetti et al. [28]. However, in our study, the statistical significance of *Enterococcus* spp. prevalence was not demonstrated, probably due to the much larger number of patients included in the analysis compared to the abovementioned study. As a possible reason for the increase in the number of BSIs caused by *Enterococcus* spp., researchers report the initial colonization of the respiratory tract of mechanically ventilated patients and then the occurrence of BSIs as a result of a worsening of the clinical condition [28].

Procalcitonin (PCT), a precursor of the hormone calcitonin, is an important inflammatory biomarker that is synthesized mainly in response to bacterial infections, reflecting the extent of the systemic inflammatory response. Bacterial toxins are crucial in the induction of the synthesis of this protein, which is also stimulated by high levels of tumor necrosis factor (TNF-α) and interleukin-6 (IL-6), produced in response to infection [29]. Procalcitonin is considered a useful diagnostic marker, allowing to distinguish bacterial infection from viral infection and as a prognostic determinant of the severity of infection and effectiveness of applied antibiotic therapy [30]. Initially, for severely ill patients with COVID-19, procalcitonin levels were used to assess the probability of secondary bacterial infection development. Since the standard treatment protocol for COVID-19 patients in critical condition has been accompanied by immunomodulators such as dexamethasone or tocilizumab (an IL-6 inhibitor), the usefulness of inflammatory biomarkers in predicting secondary bacterial infections has been unclear and has required further research.

In a research paper published in Critical Care assessing the impact of immunomodulatory therapy on the kinetics of PCT and CRP in COVID-19 patients and their usefulness in early detection of secondary bacterial infections, it was shown that the value of these two inflammatory markers was significantly reduced in patients with immunomodulation. However, dexamethasone therapy caused only a drop in CRP values. In addition, the occurrence of the so-called “rebound effect” was observed after discontinuation of immunosuppressive therapy, but the further increase of PCT and CRP in the course of secondary bacterial infections was limited. Therefore, these biomarkers have lost their diagnostic applicability for detecting secondary infections. Prolonged suppression of both PCT and CRP levels and no “rebound effect” were observed in patients treated with tocilizumab, which has a longer half-life compared to dexamethasone. Therefore, it appears that the strong and long-term anti-inflammatory effects of immunomodulatory drugs can directly impair the production of PCT and CRP to the extent that they are no longer sufficiently induced in response to a bacterial infection [31]. In our study, a similar relationship was shown, but only in the case of levels of PCT. Patients hospitalized for COVID-19 who had a history of BSIs had significantly lower PCT levels compared to patients hospitalized for other reasons where immunomodulatory drugs were not applied. An undoubted limitation of this analysis is the lack of data on the treatment of dexamethasone or tocilizumab in patients included in the study, as well as the use of levels of inflammatory markers obtained only on the day of blood sample collection for microbiological diagnostics without observing the kinetics of PCT in subsequent days. In the light of previous literature reports, we suspect that low PCT values in patients with SARS-CoV-2 infection were due to the immunosuppressive therapy that was used concomitantly, which became at some point the standard of care in the case of COVID-19 patients [31].

The analysis in terms of BSI etiology and the level of inflammatory parameters revealed another important conclusion. BSIs with the etiology of Gram-negative bacteria, including *Enterobacterales* but also *P. aeruginosa* or *A. baumannii*, have been shown to have significantly higher PCT values and LAC levels than those observed in cases of Gram-positive-caused BSIs, regardless of the occurrence of COVID-19 disease. For PCT, this dependence was also observed when the analysis was limited to patients with COVID-19 only. These observations are consistent with other scientific outcomes and are the consequence of differences in inflammatory cascade initiation between Gram-positive and Gram-negative bacteria. Gram-negative bacteria lipopolysaccharide is the main antigen that activates neutrophils through TLR-4 receptors, while the lipoic acids of Gram-positive bacteria, stimulate immune cells by TLR-2 receptors. During an infection caused by Gram-negative bacteria, the synthesis of proinflammatory cytokines such as TNF-α and IL-1, IL-6, IL-8, and IL-10 is also more frequent [32]. In addition, in the case of BSIs caused by Gram-negative rods, septic shock is much more common, which is the result of a multi-organ dysfunction caused by a generalized inflammatory reaction. This bacteremia is associated with a severe course and clinical implications and is characterized by high levels of inflammatory parameters [33].

In Polish healthcare units, the functioning of the team for the hospital antibiotic policy is a requirement imposed by the regulation of the Minister of Health of 2010, in which the head of the healthcare facility is obliged to control, among others, the scope of assessing the correctness and effectiveness of prophylaxis and antibiotic therapy. Due to the growing resistance of microorganisms to antibiotics and the need for rational antibiotic use, guidelines created by Antimicrobial Stewardship (AMS) teams should be based on local microbiological results.

The AMS in the 4th Military Clinical Hospital in Wrocław, where the study was carried out, has been operating for four years. Its task is to create empirical treatment guidelines, conduct consultations, and help optimize antimicrobial treatment. The AMS’s analysis of microbiological maps showing the local epidemiological situation from the years before the COVID-19 pandemic did not show an increased frequency of MDRO isolation. The consumption of antibiotics, including broad-spectrum antibiotics, also remained similar. Only the data collected during the COVID-19 pandemic revealed a significant increase, both in the presence of alarm microbes and in the consumption of antibiotics. The results of this study are extremely useful in the context of updating the internal guidelines. Initially, infectious disease specialists, in their recommendations for the treatment of patients with COVID-19, recommended prophylactic use of third-generation cephalosporins to reduce the risk of bacterial superinfection in the course of viral disease. These practices directly contributed to the increase in the frequency of strains producing ESBL, and the necessity to use carbapenems in the treatment of infections of this etiology resulted in a sudden increase in the isolation of carbapenemase-producing microorganisms (CPE). In addition, the health service’s problems resulting from its overload during the pandemic prevented proper isolation or cohorting of patients colonized with MDRO, which contributed to the outbreak of local mono-focal epidemics.

Moreover, based on the results obtained, it is clear that no increase in BSI caused by VRE strains has been observed in the hospital, nor has a single case of vancomycin-resistant staphylococci been detected. At the same time, MRSA still accounts for many blood infections. According to the obtained results, vancomycin is still the drug of choice for suspected gram-positive infections or empirical antibiotic therapy in septic shock and sepsis. In addition, due to the results obtained, where a significant increase in BSI caused by CPE rods was demonstrated, the emphasis on a thorough analysis of the patient’s medical history and the results of previous cultures, including the results of CPE carrier status, was increased. The standard of therapeutic management in a patient with a suspected infection caused by bacteria-producing NDM metallo-beta-lactamase was also established.

Studying the local situation and the etiological factors of BSI, their variability, and drug susceptibility has positively reduced the use of antibiotics, including carbapenems, and has helped AMSs teams update guidelines.

## 5. Conclusions

In this single-center clinical study focused on BSIs in patients before and during COVID-19, an increased frequency of BSIs was shown for COVID-19 patients. Moreover, in this group, a high rate of MDR strain isolation has been described. This was most likely due to the problems faced by health care units at that time, which concerned shortages among medical staff, severe working conditions, and a large number of patients admitted to hospitals. In addition, a significant increase in antibiotic consumption, especially those with a broad spectrum of activity, contributed to the induction of selection pressure and caused the rapid spread of MDR species such as NDM or MRSA in the hospital environment. All these factors, in combination with severe clinical conditions and overlapping secondary bacterial infections, resulted in increased mortality in the COVID-19 patient population. Due to the use of immunosuppressive therapy in patients with respiratory failure in the course of SARS-CoV-2 infection, the diagnostic value of proinflammatory markers such as PCT in the early detection of secondary bacterial infections may be limited.

## Figures and Tables

**Table 1:** General data about hospitalization within the analyzed periods.

	preCOVID-19	COVID-19
Number of hospitalizations	46,849	36,733
Patient days, n	262.174	233.447
Number of patients with blood culture performed, n	1836	2452
Number of patients with positive blood culture, n (%)	298 (16)	479 (20)
Number of patients with negative blood culture, n (%)	1297 (71)	1557 (64)
Number of patients with possible contamination in blood culture, n (%)	241 (13)	386 (16)
Incidence (BSI/1000 patient days)	1.13	2.05

**Table 2:** Frequency of co-existing diseases and blood biochemistry results in patient groups under study (A—patients hospitalized for COVID-19 or history of COVID-19 disease in the last 6 months; B—patient’s hospitalization unrelated to COVID-19; *p*—statistical significance; the *p*-value refers to a comparison of the pre-COVID-19, group A, and group B; ^C^—Chi-squared test; ^K^—Kruskal–Wallis test).

Patient Characteristics	preCOVID-19 n = 298	COVID-19 n = 479			H/χ^2^	*p*
		A n = 174	B n = 305	A + B n = 479		
Age, median (IQR), years	73 (63.3–81)	73 (66–81)	74 (64–83)	73 (65–82)	0.611	0.737 ^K^
Males, n (%)	168 (57)	106 (61)	182 (60)	288 (60)	1.078	0.583 ^C^
Diabetes, n (%)	71 (24)	62 (36)	87 (29)	149 (31)	7.556	0.023 ^C^
Chronic kidney disease, n (%)	31 (10)	37 (21)	62 (20)	99 (21)	13.965	<0.001 ^C^
Chronic cardiovascular disease, n (%)	155 (52)	54 (31)	97 (32)	151 (32)	32.334	<0.001 ^C^
Cancer, n (%)	46 (15)	21 (12)	47 (15)	68 (14)	1.213	0.543 ^C^
Obesity, n (%)	30 (10)	23 (13)	30 (10)	53 (11)	1.520	0.468 ^C^
Death, n (%)	124 (42)	103 (59)	121 (40)	224 (47)	17.644	<0.001 ^C^
WBC [×10^3^/μL], median (IQR)	13.5 (9.5–17.8)	13.8 (8.9–21)	13.3 (8.3–19.9)	13.4 (8.7–20.4)	0.749	0.688 ^K^
NEUTR [×10^3^/μL], median (IQR)	11.71 (7.56–16.01)	12.4 (7.93–18.71)	11.09 (6.41–17.15)	11.47 (6.85–17.96)	2.062	0.357 ^K^
CRP [mg/L], median (IQR)	163.5 (102–228)	159.9 (108.1–240.9)	168.1 (108.4–252.5)	164.4 (107.9–246.3)	1.594	0.451 ^K^
PCT [ng/mL], median (IQR)	5.87 (0.88–30.36)	2.35 (0.43–10.82)	4.62 (0.9–21.32)	3.43 (0.78–16.62)	14.613	<0.001 ^K^
LAC [mmol/L], median (IQR)	2.2 (1.5–3.68)	2.1 (1.45–2.85)	2.35 (1.4–4.4)	2.25 (1.4–3.93)	1.848	0.397 ^K^

**Table 3:** Species distribution among patients diagnosed with BSI, with or without COVID-19 (A—patients hospitalized for COVID-19 or history of COVID-19 disease in the last 6 months; B—patient’s hospitalization unrelated to COVID-19; *p*—statistical significance; the *p*-value refers to a comparison of the pre-COVID-19 and COVID-19 (A + B) using the Chi-squared test; NT—not tested).

Species Structure	Number (%) of Isolates *				
	preCOVID-19	COVID-19			p **
		A	B	A + B	
**Gram (+)**	**184 (60.5)**	**111 (59.4)**	**178 (56)**	**289 (57.2)**	**0.63**
*Enterococcus faecalis*	13	12	16	28	0.44
*Enterococcus faecium*	13	11	10	21	0.98
*Enterococcus gallinarum*	0	2	0	2	NT ^#^
*Staphylococcus aureus*	101	45	87	132	0.07
CoNS *	35	38	51	89	0.023
*Staphylococcus epidermidis*	20	24	36	60	0.009
*Listeria monocytogenes*	0	0	1	1	NT ^#^
*Streptococcus agalactiae*	3	0	1	1	
*Streptococus dysgalactiae*	3	0	1	1	
*Streptococcus pneumoniae*	8	1	4	5	0.56
*Streptococcus pyogenes*	2	0	0	0	
*Streptococcus* spp.	6	2	7	9	
**Gram (−)**	**107 (35.2)**	**67 (35.8)**	**128 (40.3)**	**195 (38.6)**	**0.90**
*Enterobacterales*	92 (86)	58 (86.6)	112 (87.5)	170 (87.2)	0.399
*Escherichia coli*	57	26	61	87	0.736
*Enterobacter cloacae*	5	3	11	14	0.27
*Klebsiella pneumoniae*	18	22	23	45	0.095
*Leclerciaade carboxylata*	0	0	1	1	NT ^#^
*Morganella morgannii*	0	0	5	5	NT ^#^
*Proteus mirabilis*	10	4	8	12	NT ^#^
*Salmonella* Enteritidis	0	1	1	2	NT ^#^
*Serratia marcescens*	2	2	2	4	NT ^#^
**Non-fermentative bacilli**	**15 (14)**	**9 (13.4)**	**16 (12.5)**	**25 (12.8)**	**0.909**
*Acinetobacter baumannii*	7	6	6	12	0.89
*Pseudomonas aeruginosa*	8	3	9	12	0.87
*Stenotrophomonas maltophilia*	0	0	1	1	NT ^#^
**Yeast-like fungi**	**9 (3)**	**8 (4.3)**	**7 (2.2)**	**15 (3)**	**0.53**
*Candida albicans*	5	7	3	10	0.686
*Candida glabrata*	4	1	1	2	NT ^#^
*Candida* spp.	0	0	3	3	NT ^#^
**Anaerobic**	**4 (1.3)**	**1 (0.5)**	**5 (1.6)**	**6 (1.2)**	**NT ^#^**
*Bacteroides* spp.	4	0	4	4	NT ^#^
*Fusobacterium nucleatum*	0	1	0	1	NT ^#^
*Veilonella* spp.	0	0	1	1	NT ^#^
**Total number of isolates**	**319**	**196**	**334**	**530**	

* percentages are for subgroups; ** *p* refers to all tested isolates; ^#^ not tested due to a small sample size.

**Table 4:** Distribution of antibiotic-resistant microorganisms in patients diagnosed with BSI, with or without COVID-19 (A—patients hospitalized for COVID-19 or with a history of COVID-19 disease in the last 6 months; B—patients hospitalized unrelated to COVID-19). *p*—statistical significance; the *p*-value refers to a comparison between the defined groups using the Chi-squared test.

Pathogen	Number (%) of Isolates				*p*	
	preCOVID-19	COVID-19			A vs. B	preCOVID-19 vs. A + B
		A	B	A + B		
Gram (+)						
MRSA/MSSA + MRSA	34/101 (34)	22/45 (49)	23/87 (26)	45/132 (34)	0.009	0.945
MRCNS/MSCNS + MRCNS	31/35 (89)	32/38 (84)	38/51 (75)	70/89 (79)	0.269	0.201
VRE/VRE + VSE	9/26 (35)	5/25 (20)	4/26 (15)	9/51 (18)	0.665	0.096
Gram (−)						
ESBL/Gram (−)	25/107 (23)	15/67 (22)	26/128 (20)	41/195 (21)	0.735	0.638
NDM/Gram (−)	1/107 (0.9)	11/67 (16)	3/128 (2)	14/195 (7)	0.0003	0.017

(X)—% of resistant strains within isolated species. ESBL—Gram-negative rods producing extended-spectrum beta-lactamases. MSCNS—methicillin-sensitive coagulase-negative *Staphylococcus* spp.; MRCNS-methicillin-resistant coagulase-negative *Staphylococcus* spp.; MSSA—methicillin-sensitive *S. aureus*. MRSA-methicillin-resistant *S. aureus*; NDM—Gram-negative rods producing New Delhi metallo-β-lactamases; VRE-Vancomycin resistant *Enterococcus* spp.

## Data Availability

Derived data supporting the findings of this study are available from the first author Natalia Słabisz upon request.

## Supplementary Materials

The following supporting information can be downloaded at: https://www.mdpi.com/article/10.3390/jcm12185942/s1, Table S1: Association between occurrence of particular comorbidities, number of pathogens causing BSI, and mortality—comparison between pre-COVID-19 and COVID-19 periods. P—statistical significance; the *p*-value refers to a comparison of the preCOVID-19, group A, and group B using the Chi-squared test; Table S2: Relation between causative blood-stream pathogen and blood biochemistry results. The *p*-value was calculated using the Mann–Whitney test. Status: 1/0—pathogen presence/pathogen absence.
