# Supplementary material for: Impact of the COVID-19 Pandemic on the Epidemiology of Bloodstream Infections in Hospitalized Patients—Experience from a 4th Military Clinical Hospital in Poland
Source: J Clin Med. 2023 Sep 13;12(18):5942. doi: 10.3390/jcm12185942 (PMC10531964; doi:10.3390/jcm12185942)
Supplement: Supplementary file 1 [file jcm-12-05942-s001.zip › jcm-2566596-supplementary.pdf]

Journal of
Clinical Medicine

MDPI

Supplementary

Impact of the COVID-19 Pandemic on the Epidemiology of Bloodstream Infections in Hospitalized Patients—Experience from a 4th Military Clinical Hospital in Poland

Natalia Słabisz [1], Ruth Dudek-Wicher [2,*], Patrycja Leśnik [3], Jacek Majda [1], Krzysztof Kujawa [4] and Urszula Nawrot [2]

[1] Department of Laboratory Diagnostic, 4th Military Clinical Hospital in Wroclaw, 53-114 Wroclaw, Poland; nataliaslabisz@gmail.com (N.S.); jmajda@4wsk.pl (J.M.)
[2] Department of Pharmaceutical Microbiology and Parasitology, Faculty of Pharmacy, Wroclaw Medical University, 50-367 Wroclaw, Poland; urszula.nawrot@umw.edu.pl
[3] Clinical Department of Anesthesiology and Intensive Care, 4th Military Clinical Hospital in Wroclaw, 53-114 Wroclaw, Poland; patrycja.lesnik@gmail.com
[4] Statistical Analysis Centre, Wroclaw Medical University, 50-368 Wroclaw, Poland; krzysztof.kujawa@umw.edu.pl
* Correspondence: r.dudek.wicher@gmail.com

Table S1. Association between occurrence of particular comorbidities, number of pathogens causing BSI and mortality- comparison between preCOVID-19 and COVID-19 periods. P – statistical significance; the p-value refers to a comparison of the preCOVID-19, group A, and group B using Chi-squared test

Patient characteristics	Number of patients/number of deaths				*p*
	preCOVID-19 n=298	COVID-19 n=479			
		A n=174	B n=305	A+B n=479	
**Diabetes 220/172**	71 /30	62 /43	87/28	149/71	0.506
**Chronic kidney disease, n (%)**	31 /12	37/26	62 /30	99 /56	0.079
**Chronic cardiovascular disease, n (%)**	155/77	54 /36	97 /49	151 (85)	0.041
**Cancer, n (%)**	46 /21	21 /15	47/24	68 /39	0.057
**Obesity, n (%)**	30/8	23 /10	30 /11	53 /21	0.044
**BSI due to 1 species**	292/121 (41.4)	161/93 (57.7)	292/114 (39.0)		0.028
**BSI due to 2 species**	6/3 (50%)	14/7 (50)	13/10 (76)		

Table S2. Relation between causative blood-stream pathogen and blood biochemistry results. The P-value calculated using Mann-Whitney test. Status: 1/0 – pathogen presence/pathogen absence.

**Variable**		**Gram-negative**	***Enterobacterales***	**Gram-positive**	**CoNS**	***Candida* spp.**
		Median [Q1;Q3]	Median [Q1;Q3]	Median [Q1;Q3]	Median [Q1;Q3]	Median [Q1;Q3]
WBC	1	14.1 [9.0; 21.8]	14 [8.7; 21.8]	13.2 [9.1; 18.4]	11.4 [7.8; 16.5]	11.1 [9.0; 17.2]
	0	13.1 [9.1; 18.3]	13.1 [9.1; 18.4]	13.8 [9.0; 21.7]	13.8 [9.4; 20.1]	13.5 [9.1; 19.5]
	P	0.109	0.156	0.178	0.001	0.333
NEUTR	1	12.1 [6.8; 18.8]	12.3 [6.8; 18.9]	11.3 [7.4; 16.7]	9.5 [5.7; 13.0]	10 [7.4; 14.9]
	0	11.2 [7.4; 16.5]	11.2 [7.4; 16.5]	11.8 [7.0; 18.7]	12.1 [7.4; 17.9]	11.6 [7.2; 17.4]
	P	0.229	0.246	0.543	0.000	0.349
CRP	1	176.2 [109.8; 242.9]	175.2 [109.5; 240.0]	159.7 [103; 234.7]	136.5 [74.0; 181.4]	136 [103.3; 263.1]
	0	156.3 [103.3; 234.7]	159.9 [104; 236.0]	171.1 [109.8; 242.4]	169 [111.0; 246.8]	165 [106.0; 236.6]
	P	0.055	0.161	0.118	0.000	0.565
PTC	1	14.2 [2.4; 42.3]	15.6 [2.6; 42.5]	2.3 [0.6; 9.9]	0.8 [0.2; 3.4]	2.5 [0.5; 15.5]
	0	2.2 [0.5; 9.4]	2.3 [0.6; 10.5]	12.2 [2.0; 39]	6.1 [1.0; 26.4]	4.2 [0.8; 20.9]
	P	0.000	0.000	0.000	0.000	0.334
LAC	1	2.7 [1.6; 5.3]	2.9 [1.7; 5.6]	2.1 [1.4; 3.3]	1.6 [1.1; 2.2]	2.1 [1.0; 11.1]
	0	2.1 [1.4; 3.4]	2.1 [1.4; 3.2]	2.7 [1.6; 5.2]	2.4 [1.6; 4.1]	2.2 [1.5; 3.9]
	P	0.012	0.002	0.024	0.000	0.879
